# Prenatal diagnosis of diphallia in association with bladder exstrophy: a case report

**DOI:** 10.1186/s12884-022-04746-4

**Published:** 2022-05-24

**Authors:** Homeira Vafaei, Shohreh Roozmeh, Ali Bahador, Maryam Zare Khafri, Mozhde Ghiasi

**Affiliations:** 1 Maternal-fetal medicine Research Center, Department of Obstetrics and Gynecology, School of Medicine, Shiraz University of Medical Sciences, Shiraz, Iran; 2 Maternal-Fetal Medicine Research Center, Shiraz University of Medical Sciences, Shiraz, Iran; 3 Shiraz Transplant Center, Abu Ali Sina Hospital, Shiraz University of Medical Sciences, Shiraz, Iran; 4 Dr vafaei perinatology center, Shiraz University of Medical Sciences, Shiraz, Iran

**Keywords:** Diphallia, Penile duplication, Fetal abnormalities, Prenatal diagnosis

## Abstract

**Background:**

Penile duplication or diphallia is a rare congenital anomaly with unclear pathophysiological cause. Most cases of diphallia are reported postnatally; however, today with the use of a high-resolution ultrasound device, in-uterine diagnosis of many congenital anomalies is possible.

**Case presentation:**

Herein we report a multiparous mother at 25 weeks of gestation who referred due to an abnormal cystic structure protruding from a large abdominal wall defect located below the umbilicus that was noted during a routine exam. Target scan revealed two penile-like protrusions with an empty scrotal sac and double bladder in an otherwise normal fetus, which was confirmed postnatally. Neonatal microarray study and karyotype were normal.

**Conclusion:**

Diphallia could be detected prenatally as an isolated anomaly, associated with caudal duplication syndrome, or as an exstrophy-epispadias complex. As this is a rare congenital anomaly, all sonographers should be familiar with prenatal ultrasound features and associated anomalies, an important issue in prenatal counseling with parents, delivery planning, psychological support of the family, and postnatal management.

## Background

Penile duplication, or diphallia, is a rare congenital anomaly with a frequency of 1 in 5,000,000 neonates in the United States [1]. The exact pathophysiological cause of this phenomenon is unclear; however, it seems that some teratogenic factors may interfere with the normal embryological development of the genitourinary system between the 23rd and 25th days of gestation [2, 3].

During the embryology period, columns of mesoderm at the end of the urogenital sinus around the lateral aspect of the cloacal palate grow to form the genital tubercle. Failure in the fusion of the bands of these mesoderm causes bladder exstrophy and epispadias. Consequently, for embryological occurrence of penile duplication, longitudinal duplication of the cloacal membrane should occur to form the 3 or 4 columns of primitive mesoderm around the 2 cloacal membranes to finally form the two genital tubercles [3, 4].

Although diphallia may appear as an isolated anomaly, it may also be associated with caudal duplication syndrome or as an exstrophy-epispadias complex. It may also be associated with multiple congenital malformations such as ventral herniation, musculoskeletal anomalies, anorectal malformations, and heart abnormalities [5].

Based on new clinical classification by Lisieux E groups, four types of penile duplication can be observed with various outcomes: [6].

1. True diphallia (described as 2 separated penises with 2 corpora and one spongiosum body in each one, which is usually associated with caudal duplication syndrome).
2. Hemiphalluses (with 2 halves of the penile shaft, each one containing the hemiglans and a corpus; this type is a part of exstrophy-epispadias complex).
3. Pseudodiphallia (known by a non-functional extraphallic tissue, associated with a true typical phallus).
4. Partial duplication of the penile shaft or glans.

Most cases of diphallia have been reported postnatally; however, today high-resolution ultrasound has made possible the in-utero diagnosis of many congenital anomalies. To the best of our knowledge, there exists just two cases of prenatal diagnosis of diphallia in the literature, and the present case is the third one [7, 8].

## Case presentation

The patient was a 40-year-old, multiparous woman with no comorbidity, who referred to our center for prenatal counseling at 25 weeks of gestation due to abnormal cystic structure protruding from a large abdominal wall defect located below the umbilicus observed during a routine exam. The patient was in the low risk group in the first trimester screening test. The parents were consanguine and had one normal boy.

**Fig. 1:**
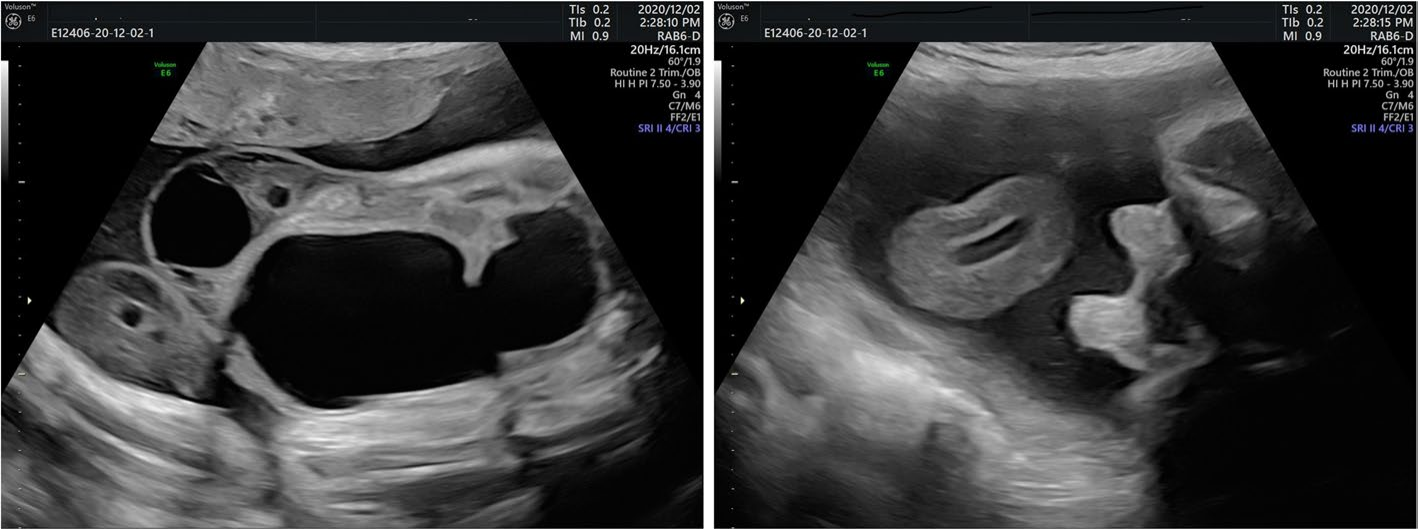
Ultrasound image at 32 weeks; large cystic structure disappeared. Large abdominal wall defect clearly seen

At the time, our scan revealed an elongated cystic lesion, measuring 85*45*65 mm which originated from the abdomen and extended to outside of the abdominal cavity with edematous umbilical cord and small deviated bladder to the left side. There was moderate hydronephrosis on the right side with renal cortical thinning. The left kidney looked normal. The bladder was small, and although the left umbilical artery was seen properly, the right one was very narrow. Evaluation of the fetal genitalia showed two penile-like protrusions with an empty scrotal sac. The fetus was otherwise normal (Fig. 1, video). For better anatomical evaluation, fetal magnetic resonance imaging (MRI) was requested and showed a large cystic structure above the normal urinary bladder, compressing it inferiorly with a large anterior abdominal wall defect, and the mentioned cystic structure extended all the way to the cord; the possibility of uracal cyst with left side pyelectasis was present due to the pressure effect of the cyst. Amniocentesis was recommended, but the parents did not agree to submit to it.

**Fig. 2:**
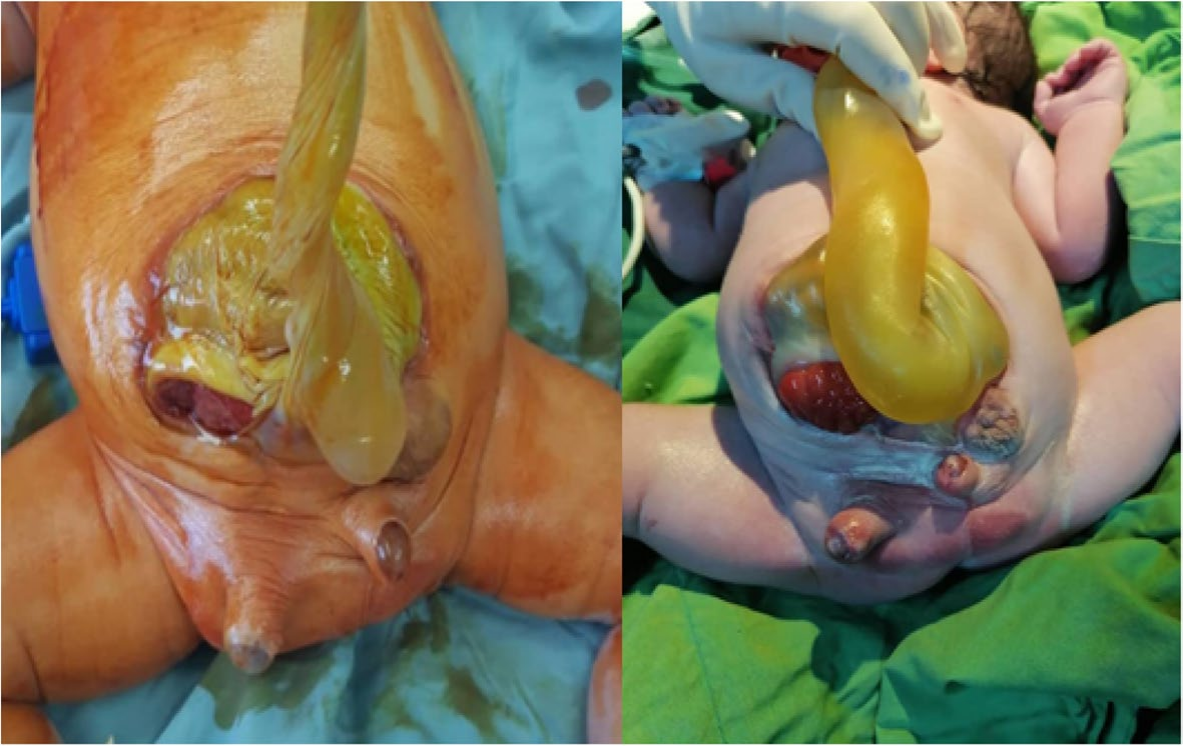
Post-delivery examination revealed a large abdominal wall defect, exomphalos. Diphallia with empty scrotum

The patient underwent serial monthly ultrasound examinations. The protruding cystic structure disappeared at the 32-week scan with an anterior abdominal wall defect. Although the fetal growth velocity and Doppler assessment were normal, there was mild polyhydramnios. It was supposed that the cystic structure had ruptured spontaneously and collapsed. The patient had planned to deliver at 38 weeks of gestation at a tertiary, fetal-maternal center with a fetal surgeon, neonatologist, perinatologist, and NICU; however, rupture of the membrane occurred at the 37th gestational week, and the patient underwent an emergency cesarean section. The boy was delivered with an APGAR score of 10 at 5 minutes and a birth weight of 3800 g. Initial examination after birth revealed a large abdominal wall defect and exomphalos measuring 80*50 mm with 2 separate phalluses in the coronal plane on the right and left sides (Fig. 2).

**Fig. 3:**
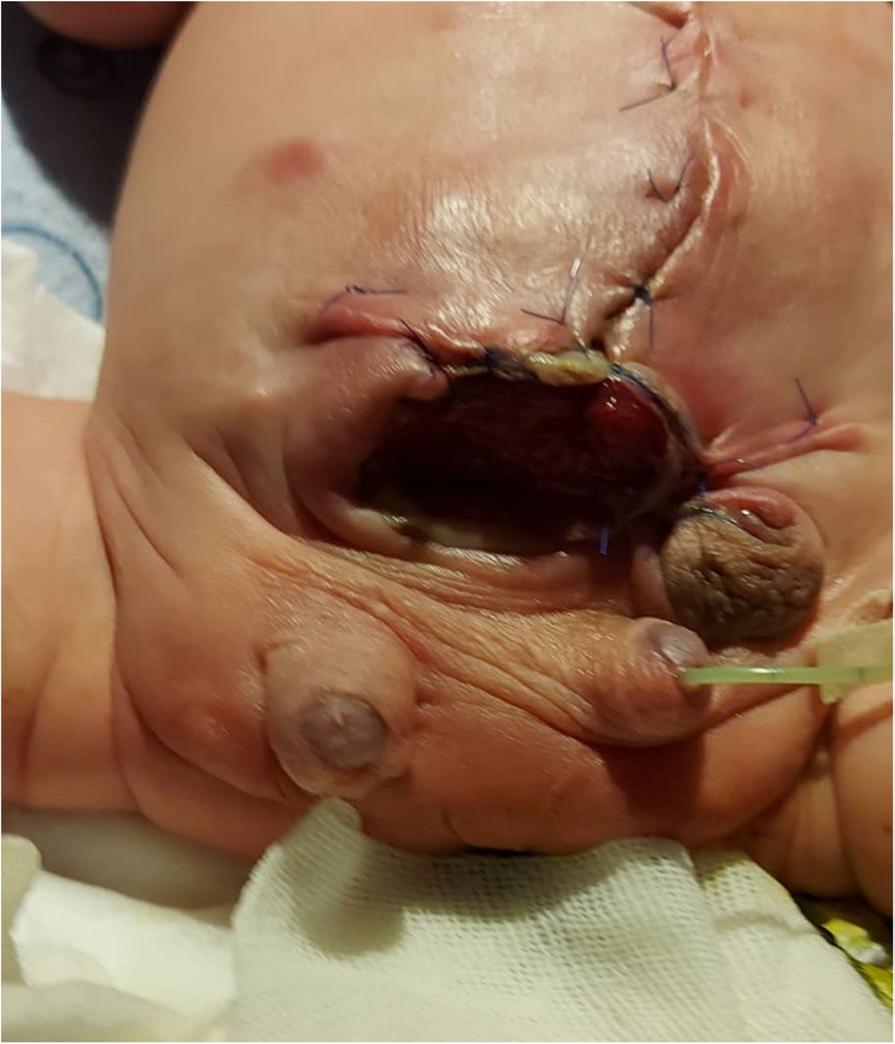
Features of post-abdominal reconstructive surgery

The baby was transferred into emergency surgery with a pediatric surgeon for closure of the abdominal wall defect. During the surgery, a complete double bladder and urethra was noted. The right bladder was exstrophic and connected to the blind end of the right urethra; there was another intra-abdominal bladder, which was connected to the normal and functional urethra at the concordant phallus. Both testes were visualized in the pelvic cavity and undescended. The anus and rectum were normal. Therefore, abdominal wall reconstruction was done using mesh (Fig. 3). Complete reconstructive surgery and orchiopexy was postponed so as to perform advanced imaging and a complete evaluation of the genitourinary system. Unfortunately, before reconstruction surgery could take place, the baby died from pneumonia and sepsis. Neonatal microarray study and karyotype were normal.

## Discussion and conclusion

Based on clinical classification by Lisieux E groups [6], the case described herein was categorized in the true diphallia group, which is commonly associated with double bladder and caudal duplication syndrome; however, in this case, the anus and colon were normal. It was thought that the large cystic structure seen in the prenatal ultrasound, which disappeared during prenatal follow-up, was one of the bladders in the diagnosis of double bladder. This bladder ruptured and disappeared during pregnancy and presented as a bladder exstrophy postnatally.

- Previously, Alireza Sina et al. reported the in-utero diagnosis of true diphallia with accessory scrotum in an otherwise normal fetus during routine anomaly scan. Examination after delivery revealed a non-functional urethral meatus on the dorsal penis with empty accessory scrotal sac, whereas there was a normally functioning ventral penis like a healthy normal 46XY karyotype baby [7].

Another case report of prenatal diagnosis of diphallia was described by Yi-An Tu et al. in a dichorionic twins pregnancy at the 23rd week of gestation with normal XY karyotype in the amniocentesis which, unfortunately, died in the uterus near term [8].

In all these cases as well as the case reported herein, fetal karyotype was normal XY. Diphallia could be detected prenatally as an isolated anomaly, associated with caudal duplication syndrome, or as an exstrophy-epispadias complex. Exact classification of this anomaly, however, is not possible in all cases. If an extra penile-like protrusion is detected during routine fetal ultrasound examination, it seems necessary to implement a target scan to detect any associated extragenital anomalies. The final prognosis of diphallia is dependent on the type of congenital anomaly and individualized treatment is based on it.

Although diphallia is a rare congenital anomaly, all sonographers should be familiar with prenatal ultrasound features and the congenital anomalies associated with diphallia. Despite the fact that the majority of children with isolated diphallia have a favorable postnatal outcome, prenatal diagnosis is important in terms of prenatal counseling with the parents and psychological support of the family. In terms of prenatal counseling with the parents, true diphallia seems to be highly associated with other severe congenital malformations compared with isolated bifid phallus, and those infants born with other congenital renal and/or colorectal anomalies have higher neonatal mortality due to various infections and sepsis, as seen in our case. It is also important for multidisciplinary team delivery planning in a tertiary center. It is also more challenging for future reconstruction surgery outcomes in the sense of continence preservation, erectile dysfunction, and external genital aesthetics.

### Patient perspective

“I am very thankful for the timely diagnosis and regular follow-up during the perinatal period, and I am also very pleased with the multidisciplinary management, timely delivery, and postnatal care. Although unfortunately I lost my baby, all medical staff did their best regarding medical and psychological support,” (translated from patient’s comments in Persian language).

## Data Availability

All data obtained during the current study are available from the corresponding author on reasonable request.

## Abbreviations

MRI: Magnetic resonance imaging
